# Movement as Medicine for Type 2 Diabetes: protocol for an open pilot study and external pilot clustered randomised controlled trial to assess acceptability, feasibility and fidelity of a multifaceted behavioural intervention targeting physical activity in primary care

**DOI:** 10.1186/1745-6215-15-46

**Published:** 2014-02-03

**Authors:** Leah Avery, Falko F Sniehotta, Sarah J Denton, Nick Steen, Elaine McColl, Roy Taylor, Michael I Trenell

**Affiliations:** 1 Institute of Cellular Medicine, William Leech Building, Faculty of Medical Sciences, Newcastle University, Newcastle upon Tyne, UK; 2 Newcastle Health Psychology Group, Institute of Health & Society, Baddiley-Clark Building, Faculty of Medical Sciences, Newcastle University, Newcastle upon Tyne, UK; 3 Institute of Health & Society, Baddiley-Clark Building, Faculty of Medical Sciences Newcastle University, Newcastle upon Tyne, UK; 4 Newcastle Clinical Trials Unit, Institute of Health & Society, William Leech Building, Faculty of Medical Sciences, Newcastle University, Newcastle upon Tyne, UK; 5 Newcastle Magnetic Resonance Centre, Campus for Ageing & Vitality, Newcastle University, Newcastle upon Tyne, UK

**Keywords:** Open pilot, Randomised controlled trial, Behaviour change, Type 2 diabetes, Physical activity, Exercise, Fidelity, Primary care

## Abstract

**Background:**

Physical activity (PA) and nutrition are the cornerstones of diabetes management. Several reviews and meta-analyses report that PA independently produces clinically important improvements in glucose control in people with Type 2 diabetes. However, it remains unclear what the optimal strategies are to increase PA behaviour in people with Type 2 diabetes in routine primary care.

**Methods:**

This study will determine whether an evidence-informed multifaceted behaviour change intervention (Movement as Medicine for Type 2 Diabetes) targeting both consultation behaviour of primary healthcare professionals and PA behaviour in adults with Type 2 diabetes is both acceptable and feasible in the primary care setting. An open pilot study conducted in two primary care practices (phase one) will assess acceptability, feasibility and fidelity. Ongoing feedback from participating primary healthcare professionals and patients will provide opportunities for systematic adaptation and refinement of the intervention and study procedures. A two-arm parallel group clustered pilot randomised controlled trial with patients from participating primary care practices in North East England will assess acceptability, feasibility, and fidelity of the intervention (versus usual clinical care) and trial processes over a 12-month period. Consultation behaviour involving fidelity of intervention delivery, diabetes and PA related knowledge, attitudes/beliefs, intentions and self-efficacy for delivering a behaviour change intervention targeting PA behaviour will be assessed in primary healthcare professionals. We will rehearse the collection of outcome data (with the focus on data yield and quality) for a future definitive trial, through outcome assessment at baseline, one, six and twelve months. An embedded qualitative process evaluation and treatment fidelity assessment will explore issues around intervention implementation and assess whether intervention components can be reliably and faithfully delivered in routine primary care.

**Discussion:**

Movement as Medicine for Type 2 Diabetes will address an important gap in the evidence-base, that is, the need for interventions to increase free-living PA behaviour in adults with Type 2 diabetes. The multifaceted intervention incorporates an online accredited training programme for primary healthcare professionals and represents, to the best of our knowledge, the first of its kind in the United Kingdom. This study will establish whether the multifaceted behavioural intervention is acceptable and feasible in routine primary care.

**Trial registration:**

Movement as Medicine for Type 2 Diabetes (MaMT2D) was registered with Current Controlled Trials on the 14th January 2012: ISRCTN67997502. The first primary care practice was randomised on the 5th October 2012.

## Background

It is estimated that 171 million people worldwide in the year 2000 had a diagnosis of Type 2 diabetes, which is predicted to rise to 366 million by 2030 [1]. Factors contributing to the increased prevalence of Type 2 diabetes include increased or unchanged calorie consumption combined with limited energy expenditure [2]. Reduced energy expenditure is important because the majority of people with Type 2 diabetes, or those at highest risk for developing the condition, are physically inactive compared with national norms [3].

Physical activity (PA)/exercise is widely considered to be an integral element of diabetes management [4]. Several reviews [5,6] and meta-analyses [4,7-9] of laboratory-based studies demonstrate that PA/exercise can produce a clinically important improvement in glucose control in people with Type 2 diabetes (in the absence of weight loss) producing an average improvement in glycated hemoglobin A1c (HbA1c) of between −0.4% and −0.6%. While this improvement is comparable to pharmacological therapies [10], pharmacology does not provide a long-term solution to glucose control in this largely progressive condition. Appropriate lifestyle behaviour changes, such as increased engagement in free-living PA, are therefore required to halt progression to subsequent classifications of drugs or insulin therapy. A 2012 meta-analysis of randomised controlled trials (RCTs) of behavioural interventions targeting free-living PA/exercise in adults with Type 2 diabetes found a clinically important improvement in HbAlc of −0.32%, (95% confidence interval (CI) = −0.44% to −0.21%) demonstrating that outcomes observed under laboratory conditions can be replicated in clinical and community settings when using evidence-based behavioural strategies [11].

The Department of Health for England currently recommends that adult’s ≥19 years old should accumulate a minimum of 150 minutes of moderate intensity PA each week to achieve tangible health benefits [12]. However, despite the known benefits of PA, there remains a shortage of effective interventions that can be offered in the primary care setting to adults with Type 2 diabetes to support them to increase their levels of PA [13]. With traditional advice-giving approaches being largely ineffective, behavioural interventions that aim to increase PA/exercise and maximise glycaemic control in the long-term are needed. Furthermore, targeting PA in the primary care setting is particularly promising because people with Type 2 diabetes have regular contact with their primary care team; therefore, multiple opportunities exist for delivery of effective interventions by primary healthcare professionals.

Although national guidelines outline the need for patient education models in the management of Type 2 diabetes [14] evidence-based tools are lacking. This is especially the case with respect to advice on effective strategies/techniques for use by diabetes care providers to increase levels of PA/exercise. The majority of people with Type 2 diabetes in the UK are offered some form of education/intervention, at least at the point of diagnosis; however, the length, information content and style of the interventions vary greatly between services. The majority of interventions do not include adequate support to help people with Type 2 diabetes to achieve PA recommendations, very few have been formally evaluated, and rarely have the individuals responsible for the delivery of these interventions been formally trained for this purpose [15]. A 2012 systematic review of behavioural interventions targeting PA/exercise reported that five out of seventeen RCTs targeting PA/exercise referred to training for care providers. Furthermore, two of these five trials explicitly described the content of training provided, and none of the included trials attempted to assess the efficacy of the training on consultation behaviour [11]. With so few primary studies explicitly utilising treatment fidelity strategies to monitor and improve training for care providers (where training is offered), or to monitor the delivery of interventions to patients in practice, it is difficult to establish whether the interventions are being delivered as intended. Therefore it becomes impossible to decipher whether reported outcomes are a function of the intervention or ‘non-intervention’ factors [16].

### Movement as Medicine for Type 2 Diabetes

The multifaceted behaviour change intervention ‘Movement as Medicine for Type 2 Diabetes’ (MaMT2D) aims to address the evidence gaps outlined above. The first component is an online training intervention programme for primary healthcare professionals to develop knowledge and skills for (1) use of a range of behaviour change counselling skills and evidence-based behaviour change techniques for improving free-living PA behaviour; and (2) delivery of a primary healthcare professional-led intervention to engage adults with Type 2 diabetes in discussions about increasing their levels of free-living PA. The second component (primary healthcare professional-led intervention) provides adults with Type 2 diabetes with evidence based intervention strategies and a range of resources to facilitate an increase in free-living PA behaviour; for example decisional balance aids, activity planners/trackers and pedometers (SW200, Yamax Corporation, Tokyo, Japan).

Following the Medical Research Council framework for the development and evaluation of complex interventions [17,18], the current intervention was developed following an initial needs assessment with patients and primary healthcare professionals. This informed a subsequent participatory development process involving primary healthcare professionals, psychologists, physiologists, clinical experts, designers and adults with Type 2 diabetes in the primary care setting. This allowed identification of the most appropriate mode of delivery and form of intervention components for patients and primary healthcare professionals.

The information content of MaMT2D was informed by the findings of a systematic review and meta-analysis of behavioural interventions targeting free-living PA/exercise in adults with Type 2 diabetes [11]. This review reported on a series of moderator analyses to identify specific candidate behaviour change techniques and intervention features associated with clinical effectiveness (≥0.3% reduction in HbA1c). Moderator analyses showed positive associations between theory-based interventions and clinical effectiveness (although no one theory held benefit over another), including interventions lasting at least six months in duration and interventions that utilised ≥10 behaviour change techniques. Analyses did not identify a specific mode of delivery (group sessions versus individual face-to-face sessions versus a combination of group and individual face-to-face sessions) associated with clinical effectiveness; therefore, mode of delivery was selected in accordance with the views elicited from primary healthcare professionals (online) and patients (individual face-to-face sessions) during the participatory development process.

Given that the optimal theoretical underpinning of behaviour change interventions targeting PA in the context of diabetes care was not elucidated by the systematic review, key selection criteria were agreed for theory selection. These criteria included a strong evidence-base for modelling the process of intervention components and outcomes that were the foci of MaMT2D (increasing free-living PA in adults with type 2 diabetes and enhancing consultation behaviour of primary healthcare professionals to deliver a PA behavioural intervention to adults with type 2 diabetes).

Exploratory work with primary healthcare professionals and patients identified the need to target: 1) motivational factors, such as attitudes/beliefs regarding the use of PA by healthcare professionals as a management option for people with Type 2 diabetes; 2) volitional factors, such as self-efficacy to increase and maintain increases in PA behaviour in adults with Type 2 diabetes; and 3) self-efficacy of healthcare professionals for delivering a behavioural intervention in routine primary care. Therefore, additional criteria for guiding theory selection included: 1) capable of targeting both motivational and volitional factors [19]; and 2) existence of in-built constructs and evidence-based strategies that can be used effectively to target both motivation/intention and actual behaviour to support maintenance. Furthermore, theories needed to have readily available, reliable and valid instruments to measure the theoretical constructs and postulated relationships between constructs to inform the evaluation strategy for MaM2TD.

In accordance with these explicit criteria, the Theory of Planned Behaviour (TPB) [20] and Social Cognitive Theory (SCT) [21] were selected to underpin the development and evaluation of the MaMT2D intervention. The TPB has been extensively and successfully used to predict intentions to increase PA and actual PA behaviour [22] including healthcare professional behaviour change [23]. However, the TPB does not provide guidance on how theoretical constructs can be targeted within interventions using evidence-based strategies, or how to address the frequent lack of concordance between motivation/intention and action, that is the ‘intention-behaviour’ gap [24].

SCT has demonstrated utility in samples of people with diabetes when predicting PA behaviour [25] and provides specific evidence-based strategies for translating motivation/intentions into action/behaviour in both healthcare professionals and patients (for example, observational learning strategies, such as modelling to support the acquisition of behaviour changes skills and self-efficacy for the effective delivery of behaviour change techniques to patients). Together the TPB and SCT provide a complimentary theoretical framework for guiding the design and evaluation of the intervention components within MaMT2D.

In addition, a 2012 systematic review and meta-analysis identified a range of evidence-based behaviour change techniques associated with clinically important improvements in glycaemic control in adults with Type 2 diabetes [11]. These techniques were included in the MaM2TD intervention alongside other evidence-based intervention components/techniques informed by TPB and SCT. Briefly they are: use of follow-up prompts (PA behaviour will be prompted at the three-month time point via a telephone call from a participating primary healthcare professional and a motivational postcard from the research team); information on where and when to perform PA behaviour (primary healthcare professionals will be given access to an online repository where they can access specific details about local activities, for example, time and location); plan social support/social change (patients will be encouraged to elicit support (practical or emotional) from friends and family members); goal setting behaviour (patients will be supported to set their own PA goals); barrier identification/problem solving (patients will be asked to consider in advance barriers to attaining their goals and encouraged to consider ways to overcome them); time management (patients will be supported to identify appropriate times to be active); prompt rewards contingent on effort or progress made towards a predetermined goal (patients will be encouraged to reward any progress made towards PA goals); prompt review of behavioural goals (PA goals will be reviewed four times over a twelve-month period); prompt generalisation of PA behaviour (patients will be supported to increase PA behaviour in a different setting or situation once they have mastered it in one setting); prompt focus on past success (patients will be asked to consider past successful attempts at PA); provide information on the consequences of behaviour to the individual (patients will receive specific and relevant information if/when requested, for example, the effect of PA on their HbA1c); feedback on performance (feedback on consequences of behaviour will be provided to patients during each review appointment); action planning (patients will be supported to set SMART goals (specific, measurable, achievable, realistic and timely) [26]; and self-monitoring of PA behaviour (patients will be offered use of activity planners, trackers and a pedometer).

The efficacy of several techniques identified by the systematic review (and others included in the current intervention) has been reported previously in the context of PA behaviour change. For example, interventions utilising action planning and coping planning were found to predict an increase in PA [27]. Indeed, it has been reported that inclusion of both action planning and coping planning at different stages of the behaviour change process is optimal when targeting PA behaviour change [28,29]. Self-regulation strategies, such as self-monitoring, are reported to be more effective than interventions not utilising this technique [30,31]. Furthermore, the effectiveness of interventions is optimised when self-monitoring is combined with at least one other self-regulatory technique [32].

The behaviour change techniques selected are consistent with theories used to integrate and organise the delivery of techniques in MaMT2D [33]. The online training intervention programme is designed to provide primary healthcare professionals with the knowledge, skills and confidence (self-efficacy) to use the MaMT2D behaviour change techniques and intervention components in practice.

Finally, primary healthcare professionals will be shown how to use a range of behaviour change strategies/counselling skills to promote PA behaviour change. These include: agenda setting; use of importance and confidence rulers to initiate change talk; a decisional balance aid to discuss pros and cons for change; and communication skills (asking open questions, active listening and informing).

This protocol describes a two-phase process that will be used to develop and evaluate MaMT2D. Phase one is an open pilot study that will assess the acceptability, feasibility and fidelity of delivery of MaMT2D and will provide opportunities for intervention optimisation and refinement of study processes and procedures. Phase two is an external pilot cluster randomised controlled trial (RCT) that will assess acceptability, feasibility, and fidelity of MaMT2D (informed by any necessary optimisation identified in phase one) over a 12 month period. A qualitative process evaluation and treatment fidelity assessment will be carried out throughout the trial period, to identify any barriers and facilitators to implementation as well as adherence to the study protocol by primary healthcare professionals and patients. The pilot RCT will be conducted and reported in accordance with the Consolidated Standards of Reporting Trials (CONSORT) [34] and the Standard Protocol Items: Recommendations for Interventional Trials (SPIRIT) guidelines [See Additional file 1: SPIRIT Checklist) [35].

### Phase One: Movement as Medicine for Type 2 Diabetes: Open Pilot Study

#### Objectives

The primary objective is to conduct an open pilot study to establish the acceptability, feasibility and fidelity of the multifaceted intervention MaMT2D in the primary care setting. Intervention components will be iteratively optimised incorporating feedback from primary healthcare professionals and patients during the open pilot study. Recruitment, retention and adherence rates, including willingness to take part in video recordings of consultations, semi structured interviews, focus group discussions and complete study outcome measures will be assessed to inform a pilot RCT (phase two).

#### Methods/design

The open pilot study (phase one) will assess and optimise the acceptability, feasibility and fidelity of intervention delivery before MaMT2D enters a pilot RCT. Due to the high level of user involvement (healthcare professionals and patients) throughout the intervention development process, it is unlikely that the open pilot study will raise any important issues. An open pilot component was incorporated to identify and resolve any implementation issues and facilitate refinements to the intervention ahead of a planned pilot RCT. This will increase the likelihood that the intervention will be acceptable, feasible and delivered to a satisfactory level of fidelity. Should the open pilot indicate that some modifications to the intervention and research procedures are needed, these will be made prior to moving onto the pilot RCT.

#### Sample size

Two primary care practices in the County Durham and Darlington area of North East England will each recruit 15 patients (n = 30) in line with standard guidance for pilot studies [36].

### Eligibility criteria for primary care practices

The eligibility criteria for primary care practices are as follows:

1. Willingness to be randomised to the intervention or control group.

2. A commitment to participate over the duration of the study (12-months).

3. Allow participation of at least two primary healthcare professionals in the study.

4. Capacity and willingness to identify and recruit patients meeting the eligibility criteria.

5. Allow access to patient records for collection of clinical data.

6. Willingness to complete the study as per the protocol.

Participating primary healthcare professionals will be all members of the primary care team. These include general practitioners, practice nurses, nurse practitioners, healthcare assistants, and allied health professionals (for example, physiotherapists and occupational therapists). Primary healthcare professionals will be excluded from the study if they are currently taking part in any behaviour change intervention research.

### Eligibility criteria for patients

The eligibility criteria for patients are as follows:

1. Aged 18 years and older.

2. A confirmed diagnosis of non-insulin dependent Type 2 diabetes for a minimum of two years.

3. Capacity to provide informed written consent (that is, as established by the primary healthcare professional responsible for the patient’s care).

4. Ability to write and converse in English (that is, patients who do not require the support of an interpreter).

Patients will be excluded if they are currently taking part in any other intervention research studies; are prescribed sulphonylureas; or have evidence of heart disease, musculoskeletal disorders or other disabling conditions that could be worsened by increasing levels of PA. A primary care data quality analyst from the North of England Commissioning Support Service will provide each primary care practice with system specific reports to enable them to identify patients who meet the eligibility criteria.

Patients registered at each participating practice who fulfil pre-defined eligibility criteria will be invited to participate in the study.

### Recruitment procedures

**Figure 1:**
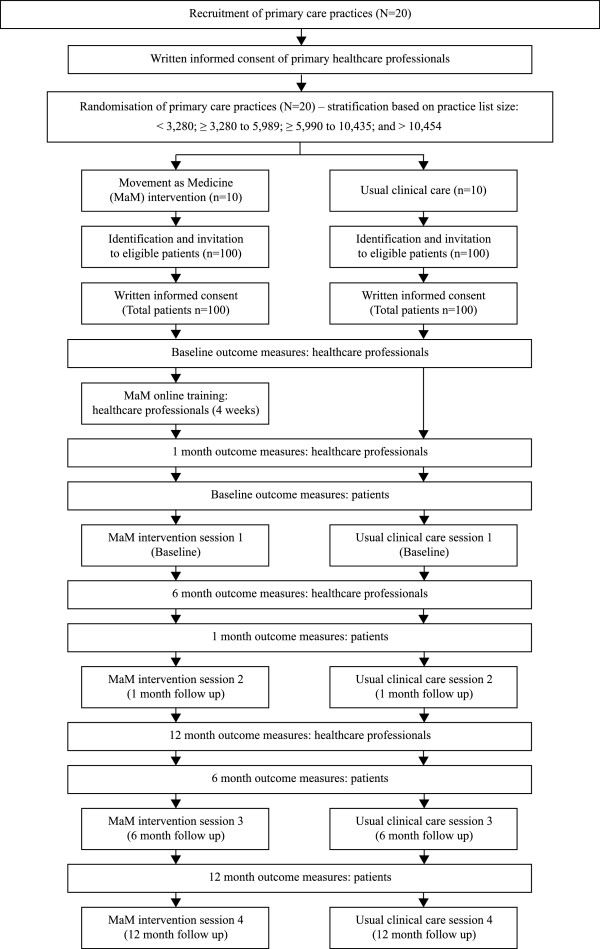
Flowchart showing the planned study recruitment processes for primary care practices, professionals and patients.

Figure 1 provides an overview of practice and patient recruitment.

All primary care practices in the County Durham and Darlington area of North East England will be invited to participate in the study. The Primary Care Research Network (PCRN) and clinical leads from the North East Commissioning Service will facilitate practice recruitment by distributing postal invitations to all practices and making personal contact with practice managers and clinical commissioning groups.

To allow continuity of care (should one primary healthcare professional withdraw from the study), a minimum of two primary healthcare professionals from each participating practice will be required to provide informed written consent to take part in the study. Consent will be sought from primary healthcare professionals to complete the online training intervention programme and subsequently to deliver the primary healthcare professional-led intervention to patients recruited to the study (should their practice be randomly allocated to MaMT2D); and to complete online study questionnaires at baseline, one, six and twelve months. Separate consent will be sought to video record consultations with patients for the purposes of treatment fidelity assessment; to take part in one or more interviews with a researcher to identify barriers and enabling factors to effective implementation of the intervention in primary care; and to take part in a focus group discussion with a researcher and other participating primary healthcare professionals upon completion of the study (to gauge overall opinion of the intervention and study processes and procedures, including ways in which they could be improved). If primary healthcare professionals decide to opt out of video recordings, interviews and focus group discussions, they can still participate in the main study. A member of the research team will obtain informed written consent from each participating primary healthcare professional [See Additional file 2: Healthcare Professional Consent Form and Additional file 3: Practice Information Sheet]. Two copies of the consent form will be signed and countersigned. One copy will be retained by the practice and stored in their study site file and a second copy retained and filed securely by the research team. The decision regarding participation is entirely voluntary and participants are free to withdraw from the study at any time. Ethical permission was not sought to collect data from participants once they have made a decision to leave the study (that is, following cessation of outcome data provision and discontinuation of treatment); although any data collected up until the point of withdrawal will be included in the analyses, unless the participant (healthcare professional or patient) specifically requests they be removed.

Specific design decisions were made to limit the amount of missing data in accordance with published guidance [37]. The MaMT2D intervention was designed to be flexible and to accommodate individual differences; therefore, patients will be supported by primary healthcare professionals to identify their activity of choice, including frequency, intensity and duration to increase tolerability. Furthermore, MaMT2D was designed to be integrated into usual clinical care. Consequently, patients randomised to the MaMT2D intervention will receive the intervention components during their usual diabetes review appointments to reduce burden. Finally, an experienced research nurse employed by the Diabetes Research Network will collect clinical outcome data from electronic patient records during each data collection phase (that is, baseline, one, six and twelve months) to minimise the burden on participating healthcare professionals and practice personnel. Information from each participant consent form will be entered into a password protected data management system that will record consent preferences (for example, decision to participate or not in semi-structured interviews). Each practice will be randomised to the intervention or control group following receipt of informed written consent by all participating primary healthcare professionals.

Practices will be stratified based on list size (with three cut-off points: <3,280; ≥3,280 to 5,989; ≥5,990 to 10,453; and >10,454) and randomised to either the intervention or control group prior to identification and recruitment of patients.

A nominated member of staff from each participating practice will be asked to identify all patients registered on their practice list who meet the eligibility criteria. An invitation letter printed on practice headed paper, a standardised patient information sheet and consent form [See Additional file 4: Patient Information Sheet Control Group; Additional file 5: Patient Information Sheet Intervention Group; and Additional file 6: Patient Consent Form] along with a pre-paid envelope will be mailed to all eligible patients. Patients who do not respond to the invitation within a six-week period will be mailed a reminder letter. Patients will be asked to provide informed written consent to attend four face-to-face appointments at their primary care practice; wear an accelerometer for seven continuous days, complete a study questionnaire and have clinical measures taken at each data collection time point (baseline, one, six and twelve months). In addition, separate consent will be sought to video record their consultations; to take part in semi-structured interviews with a researcher (to identify enabling factors and barriers to effective implementation of MaMT2D in primary care); and to take part in a focus group discussion with a researcher and other participating patients from their practice upon completion of the study (to gauge overall opinion of the intervention and study processes and procedures, including ways in which they could be improved). If patients decide to opt out of video recordings, interviews and focus group discussions, they can still remain in the main study. Consent from both the healthcare professional and patient are required before a video recording of a consultation can take place. The first two practices to provide informed written consent will enter the open pilot study described above. Each practice will be provided with a site file containing a copy of the trial protocol, patient information sheets and consent forms for reference purposes and a log for recording and monitoring patient recruitment, attrition and adverse events. If a drop-out or adverse event occurs, the contact person at the practice will be asked to contact a member of the study team to provide details. A log of all recruitment activity will be maintained by the study team and stored on a password-protected server at Newcastle University.

Participating primary healthcare professionals will be interviewed by a member of the research team prior to, and following, completion of the online training intervention programme. This training has been accredited by the Federation of the Royal College of Physicians, with completion conferring three Continuing Professional Development points. The online training intervention programme has been designed to: (1) increase the diabetes and PA related knowledge of primary healthcare professionals; (2) develop positive attitudes/beliefs towards use of PA as a management option for diabetes; (3) enhance self-efficacy for delivering a primary healthcare professional-led behavioural intervention targeting PA to patients with Type 2 diabetes; and (4) promote skills development in terms of delivering specific behaviour change techniques and behaviour change counselling skills in the context of diabetes management.

Interviews with primary healthcare professionals will focus specifically on the acceptability and feasibility of the MaMT2D intervention, including study processes and procedures. Suggestions for improvement will be discussed with the research team and, where feasible, incorporated into the intervention, research processes and procedures for the subsequent pilot randomised controlled trial. Primary healthcare professionals at participating practices will be asked to review all changes prior to their implementation. The interview topic guides will be developed using the Theoretical Domains Framework (TDF) [38]. The TDF is the result of a multi-disciplinary expert consensus approach aiming to organise and simplify the theoretical literature on behaviour change by reviewing 33 behavioural theories and organising the 128 key constructs into broad ‘theory domains’ based on commonalities [39]. After further validations, the TDF consists of 14 theory domains each providing broad explanations of why a behaviour was or was not performed: ‘Knowledge’, ‘Skills’, ‘Professional Role and Identity’, ‘Beliefs about Capabilities’, ‘Optimism’, ‘Beliefs about Consequences’, ‘Reinforcement’, ‘Intentions’, ‘Goals’, ‘Memory, Attention and Decision Processes’, ‘Environmental Context and Resources’, ‘Social Influences’, ‘Emotions’ and ‘Behavioural Regulation’ [38]. While the TDF is not a theory as such, it has been useful in organising narrative data to link findings of qualitative research back to theory [40,41] and is used here to identify domain specific issues with the online training intervention programme (for example, knowledge/skills gaps) and supporting materials for the primary healthcare professional-led intervention for use with patients.

Interviews during phase one will be conducted with patients immediately following their diabetes review appointments incorporating MaMT2D at baseline and at one-month follow-up. Patients will be interviewed by a member of the research team using a topic guide developed using the TDF to establish comprehension, acceptability of the primary healthcare professional-led intervention and to detect any domain specific issues (for example, information/support gaps).

Treatment fidelity assessment with primary healthcare professionals (video recordings of MaMT2D consultations with patients) will identify adherence to components of the primary healthcare professional-led intervention in accordance with the content of the online training intervention programme. A fidelity checklist developed for the purpose of this study will be used to assess presence/absence and appropriate use of intervention components. This will enable insights into potential adaptations or improvements to the online training intervention programme for primary healthcare professionals, including the tools (paper-based materials, DVD and pedometer) used within the primary healthcare professional-led intervention. Review appointments will be video recorded and assessed for treatment fidelity only when both participating primary healthcare professionals and patients provide their informed written consent. Following published guidance, 20% to 40% of consultations will be video recorded and, where feasible, equal numbers of consultations will be video recorded at each intervention time point to ensure representativeness of the data set [42].

Healthcare professional and patient data (including consent, fidelity, outcome data and patient appointment dates) will be entered in a data management system by a member of the research team. A data manager from the Diabetes Research Network will enter patient questionnaire data at each data collection time point. Questionnaire data from healthcare professionals will be transferred electronically from Survey Monkey by a member of the research team. The data management system will generate alerts via email ahead of each participant’s data collection time point and a member of the research team will respond to these alerts accordingly.

The use of an open pilot study enables an iterative and systematic approach to intervention adaptation and refinement, to optimise the multifaceted intervention for use in the clinical setting and to refine study processes and procedures [43].

### Amendments to intervention components

An assessment will be made following completion of the open pilot study to determine whether any amendments to the intervention and study processes and procedures are required. All interview data will be analysed by LA (and a proportion of transcripts analysed by SJD) and any issues with the intervention, study processes and procedures will be addressed following discussion with the research team. A final decision to continue to the pilot RCT will be made by the principal investigator, MIT. All video recordings from the open pilot consultations will be coded by LA (a proportion double coded by a researcher with expertise in health behaviour change to ensure reliability) and discussed with the research team. An assessment will be made as to whether fidelity of delivery (adherence to intervention components) is sufficiently high to permit the study to continue to the pilot RCT in phase two (that is, whether primary healthcare professionals have attained adequate coverage of intervention components where appropriate). Where omissions are consistent across primary healthcare professionals, reasons for this will be explored and the research team will discuss how the programme can be improved to increase fidelity during phase two. Measuring adherence to pre-defined intervention components is quantifiable; however, adherence may not require all components of an intervention to be delivered during every individual session [44]. For example, the use of the technique ‘generalisation of physical activity behaviour’ is unlikely to be used during a baseline session; therefore, appropriate absence of this technique will be recorded during the coding process. Where it is appropriate to use a behaviour change technique or an intervention component, a fidelity threshold will be applied where 50% will be considered ‘sufficiently high’ to continue to the pilot RCT in phase two.

Following amendments to the intervention, primary healthcare professionals will be asked to revisit the online training intervention programme and materials to review any revised (or new) content before continuing to deliver the intervention to patients. All amendments to the intervention and study processes and procedures will be made prior to commencement of phase two of the study. Any changes to the study protocol will be documented.

### Movement as Medicine for Type 2 Diabetes: Components of the intervention

This multifaceted behaviour change intervention comprises two distinct but interrelated components: an online training intervention programme for healthcare professionals and a primary healthcare professional-led intervention toolkit for patients.

### Healthcare professional online training intervention programme

The online training intervention programme presents eight distinct but interrelated modules that cover topics such as diabetes and metabolism, PA and exercise and the psychology of behaviour change. Module 7 is a dedicated ‘skills based’ module that aims to equip primary healthcare professionals with the skills to use pre-selected behaviour change techniques and behaviour change counselling skills listed previously to promote patient centeredness and increase PA behaviour in patients. Textual information, interactive features, video demonstrations, and consolidation quiz questions at the end of each core module aim to prepare primary healthcare professionals to confidently and faithfully deliver the primary healthcare professional-led intervention to participating patients. Once all content of the training intervention programme is viewed by primary healthcare professionals an online certificate will be generated to confirm completion.

Primary healthcare professionals employed by both primary care practices will complete the online training intervention programme component of MaMT2D. They will be emailed a unique login ID to access the password protected programme and will be given four weeks to complete all modules before using the intervention with patients. Ongoing access to the programme will be provided for the duration of the study and can be accessed at any time. Table 1 provides an overview of the MaMT2D online intervention training programme for primary healthcare professionals.

**Table 1:** Components of the online training programme and their relationship to constructs within TPB and SCT

**Module**	**Form and information content**	**Theoretical constructs**
Module 1:	Video recording of a professor of movement and metabolism introducing the programme and providing details of how and why MaMT2D was developed	Symbolising (SCT)
Introduction to MaMT2D		Attitudes and beliefs (TPB)
		Subjective norms (TPB)
	Video recording of a Consultant Diabetologist and a Diabetes Specialist Nurse providing an overview of why PA is important for the management of T2D	
Modules 3 (metabolism & type 2 Diabetes), 4 (physical activity in the care of type 2 Diabetes); and 5 (physical activity & exercise)	Evidence-based information about the role of metabolism, PA and exercise in the context of Type 2 diabetes	Symbolising (SCT)
		Attitudes/beliefs (TPB)
		Forethought (SCT)
		Intention (TPB)
Module 6:	Evidence-based information on the use of psychological theory and theory linked behaviour change techniques and counselling skills to change PA behaviour	Attitudes/beliefs (TPB)
Using psychology to change physical activity Behaviour		Forethought (SCT)
		Intention (TPB)
Module 7: Using behaviour change techniques to increase physical activity behaviour	Video demonstrations of a Diabetes Specialist Nurse demonstrating the use of behaviour change techniques and behaviour change counselling techniques in practice with a mock patient	Symbolising (SCT)
		Observational Learning (SCT)
		Perceived Behavioural Control (TPB) and Self-efficacy (SCT)
		Subjective norms (TPB)
Module 8:	Flowchart diagram demonstrating how to screen adults with T2D prior to PA/exercise	Self-regulation (SCT)
Screening before physical activity		Perceived Behavioural Control (TPB) and Self-efficacy (SCT)
End of module 3,4 and 5 quiz questions	Provides feedback on performance	Perceived Behavioural Control (TPB) and Self-efficacy (SCT)
Flowchart summary (crib sheet with prompts) of the protocol for use of the patient toolkit during diabetes review appointments	Prompts for healthcare professionals to use specific behaviour change skills and techniques	Symbolising (SCT)
		Perceived Behavioural Control (TPB) and Self-efficacy (SCT)
		Intention (TPB)
		Self-regulation (SCT)

MaMT2D, Movement as Medicine for Type 2 Diabetes; TBP, Theory of Planned Behaviour; SCT, Social Cognitive Theory. Module 2 of the online training intervention programme provided participating healthcare professionals with an overview of the research process, that is how MaMT2D will be evaluated. It was not designed to target any theoretical constructs and as such was not included in Table 1.

### Primary healthcare professional-led intervention toolkit for patients

Patients will, in addition to usual clinical care, receive the primary healthcare professional-led intervention that involves support from a trained primary healthcare professional to use a range of printed materials, DVD and pedometer (SW200, Yamax Corporation, Tokyo, Japan) to help them to increase their everyday levels of PA. The printed materials comprise: 1) a discussion card presenting a decisional balance aid, importance and confidence rulers and a seven-day PA recall diary to enable individualised feedback to be provided on current PA behaviour (type, frequency, intensity and duration); and (2) a booklet to support patients through the process of short and long-term PA goal setting, action planning, self-monitoring towards their PA goal and encouragement to use rewards contingent on progress made towards a previously set PA goal. A menu of PA options is presented to encourage patient autonomy (that is, to consider what type of PA they would like to incorporate into their everyday lives), and activity planners and trackers are provided to allow patients to self-monitor the amount of time they spend active (or the number of steps taken each day) with reference to a predetermined PA goal (that is, patients will be supported to develop their own PA goals and plans and revise them based on their individual needs and preferences). Table 2 provides an overview of the content of the practitioner-led intervention.

**Table 2:** Components of the healthcare professional-led intervention and their relationship to constructs within TPB and SCT

**Intervention component**	**Form and information content**	**Theoretical constructs**
Discussion card	Assessment of PA behaviour using a seven-day recall	Attitudes/beliefs (TPB)
		Forethought (SCT)
	A decisional balance aid to assess the pros versus the cons for changing PA behaviour	Intention (TPB)
		Perceived Behavioural Control (TPB) and Self-efficacy (TPB)
	Rulers assessing importance and confidence for change	
Booklet	Support to select an appropriate PA/exercise; set PA goals; consider means of social support; identify barriers/problem solve; set short and long-term goals; plan activity; self-monitor activity; prevent relapse	Forethought (SCT)
		Subjective norms (TPB)
		Intention (TPB)
		Self-regulation (SCT)
		Perceived Behavioural Control (TPB) and Self-efficacy (TPB)
Activity planners/trackers	Means to plan and monitor PA/exercise	Self-regulation (SCT)
DVD	Video recordings of adults with T2D engaging in PA/exercise and sharing their stories	Symbolising (SCT)
		Attitudes/beliefs (TPB)
		Observational Learning (SCT)
		Subjective norms (TPB)
		Perceived Behavioural Control (TPB) and Self-efficacy (SCT)
Pedometer	Device to monitor the number of steps taken each day	Self-regulation (SCT)
Record of progress pad	Record of readiness ruler outcomes; short and long-term PA/exercise goals; social support; potential barriers and ways to overcome them; self-monitoring method adopted; and activities of choice. Provides a mechanism for provision of feedback and an opportunity to monitor progress and recap during subsequent sessions	Perceived Behavioural Control (TPB) and Self-efficacy (SCT)
		Intention (TPB)
		Self-regulation (SCT)
Diabetes UK Leaflet	Leaflet entitled: Keeping active: an essential part of managing diabetes	Attitudes/beliefs (TPB)
		Perceived Behavioural Control (TPB) and Self-efficacy (SCT)
		Intention (TPB)

MaMT2D, Movement as Medicine for Type 2 Diabetes; TBP, Theory of Planned Behaviour; SCT, Social Cognitive Theory.

Patients will be invited to attend four face-to-face appointments with a participating primary healthcare professional from their practice (baseline, one, six and twelve months). Where possible, patients will see the same primary healthcare professional throughout the duration of the study to provide continuity. During these appointments they will be given a Diabetes UK leaflet and will receive the primary healthcare professional-led intervention. During each appointment the primary healthcare professional will complete a record of progress sheet to serve as a log of decisions made during each appointment (for example, whether a patient selected a preferred activity, set a PA goal and self-monitoring method). A carbon copy of the progress sheet will be retained by the primary healthcare professional to refer to during subsequent appointments. Materials will be used with all patients during baseline and one month appointments in both practices before any necessary changes are made. Once changes are made, the updated materials will be reintroduced and the study will continue to the 12-month intervention endpoint.

Follow up prompts have shown promising effects in behaviour change interventions while targeting a range of behaviours including physical activity [11,45,46]. Therefore, patients will receive a telephone call three months from baseline from a primary healthcare professional to ‘prompt’ PA behaviour. They will be asked if they have any questions regarding use of the intervention materials and offered support where needed to set or revise PA goals and receive individualised feedback based on performance where appropriate. At this time patients will also receive a ‘motivational’ card by mail from the research team thanking them for continuing to take part in the study and encouraging them to consider ways in which they could reduce their sedentary time and increase PA behaviour.

### Analyses

The focus of phase one (the open pilot study) is acceptability and feasibility of the intervention in routine primary care. Therefore, the proportion of participating primary healthcare professionals who login to the online training intervention programme and complete it within the required time period (four weeks) will be assessed. The proportion of participating patients who attend/fail to attend MaMT2D appointments will also be assessed. Finally, completion rates of questionnaires for both primary healthcare professionals and patients will be assessed throughout the open pilot study. Where suggestions for improvement are made by primary healthcare professionals and patients during semi-structured interviews, these will be discussed by the research team and implemented where appropriate/feasible. Analyses of these data will be descriptive, with 95% CIs reported where appropriate. Analyses of qualitative data collected using the Theoretical Domains Framework will identify domain specific beliefs likely to positively or negatively influence acceptability and feasibility of MaMT2D in the primary care setting.

### Phase two: Movement as Medicine for Type 2 Diabetes: Pilot randomised controlled trial

#### Objectives

The aim is to assess acceptability, feasibility and fidelity of MaMT2D in the primary care setting over a 12-month period.

The primary objectives are:

1. To demonstrate that recruitment of primary care practices and patients to the trial is feasible; to determine the length of time required to complete practice and patient recruitment; and to assess eligibility, recruitment, retention and adherence rates and willingness to be randomised.

2. To assess whether intervention components are delivered faithfully and consistently in accordance with the protocol over a 12-month period.

3. To conduct a qualitative process evaluation to identify enabling factors and barriers to effective implementation of MaMT2D in primary care.

The secondary objectives are:

1. To estimate the variability of objectively assessed PA behaviour, diabetes related knowledge, attitudes/beliefs, self-efficacy for being physically active and health related quality of life, HbA1c, blood pressure, waist circumference and body mass index in patients by generating interval estimates of the mean difference between groups for each outcome measure.

2. To estimate the variability both between subjects and between clusters of PA related knowledge, self-reported PA behaviour, attitudes/beliefs, self-efficacy for delivering a behavioural intervention and fidelity of specific behaviour change techniques and behaviour change counselling skills in the context of diabetes management in primary healthcare professionals. Interval estimates of the mean difference between groups for each outcome measure will be generated.

Estimates of variability will include the standard deviation, range and interquartile range for each of the study outcomes. The estimates of standard deviation will be used to generate 95% CIs for mean scores in each group and the difference in group means.

## Methods/Design

The second phase will involve a two-arm parallel group cluster RCT (practices will be the unit of randomisation) to assess acceptability, feasibility and fidelity of the intervention and study procedures over a 12-month period.

### Sample size

A total of 200 patients will be recruited from up to 20 primary care practices in the County Durham and Darlington area of North East England. The final number of practices will be identified following completion of phase one (open pilot) where an assessment of practice recruitment and retention rates will be conducted, with reference to practice list size (with three cut-off points: <3,280; ≥3,280 to 5,989; ≥5,990 to 10,453; and >10,454); the number of patients fulfilling the eligibility criteria and the number of patients recruited per practice. Up to 10 practices will be randomised to MaMT2D and a further 10 to usual clinical care. Although the trial is not powered for a definitive analysis, exploratory analyses examining between group differences will be reported based on a calculation of physical activity behaviour and HbA1c. The data generated from the pilot RCT including an estimate of the intra-cluster correlation co-efficient [47] will facilitate a power calculation to inform a definitive RCT if acceptability, feasibility and fidelity are demonstrated.

### Eligibility criteria for primary care practices and patients

Eligibility criteria for primary healthcare practices and patients will be identical to those used in phase one.

### Recruitment procedures

Figure 1 presented earlier provides an overview of practice and patient recruitment.

Recruitment procedures will be identical to those used during the open pilot study and primary healthcare professionals from each participating practice will be required to provide informed written consent prior to randomisation.

### Pilot RCT trial arms

There are two arms to the trial, (1) the control group: usual clinical care; and (2) the intervention group: MaMT2D. Despite the known benefits of PA on glycaemic control, there remains a lack of evidence-based interventions designed to increase PA in people with Type 2 diabetes. Furthermore, it remains unclear what the optimal strategies are to increase PA behaviour in people with Type 2 diabetes and whether a programme such as MaMT2D will be acceptable and feasible in the primary care setting.

Figure 1 provides an overview of planned recruitment numbers, timing of the intervention sessions and outcome measures.

### Usual clinical care (control group)

Patients will be asked to attend four face-to-face appointments (baseline, one, six and twelve months). During the initial appointment they will be given a Diabetes UK leaflet (‘Keeping active: an essential part of managing diabetes’) [48]. No further information or advice will be provided to patients about PA over and above usual clinical care.

At the three-month follow-up all participating patients will receive a telephone call from a primary healthcare professional at their practice to ask if they have any questions regarding their diabetes management and participation in the study. At the same time patients will be mailed a postcard by the research team thanking them for continuing to take part in the study.

### Movement as Medicine for Type 2 Diabetes (the intervention)

#### Healthcare professional online training intervention programme

Primary healthcare professionals employed by practices randomised to the intervention arm of the study will complete the online training intervention programme component of MaMT2D (optimised following the open pilot during phase one). The procedure for access and content is described previously for the open pilot study.

Primary healthcare practices randomised to deliver usual clinical care will be offered access to the online training intervention programme following completion of the pilot RCT.

### Primary healthcare professional-led intervention

Patients will be asked to attend four face-to-face appointments (baseline, one, six and twelve months). During these appointments they will be given a Diabetes UK leaflet (also issued to the control group) and will receive the optimised version of the primary healthcare professional-led intervention and associated materials informed by findings of the open pilot in phase one. Intervention components are described previously for the open pilot study.

### Primary outcome measures

1. An assessment of primary care practices and patient’s willingness to take part in the trial will be conducted by examining the proportion of practices and patients that were both eligible and asked to participate against those who actually agreed to participate.

2. The patient identification procedure will be examined to determine whether identification of eligible patients from practice lists was feasible, rates of eligibility and reasons for ineligibility.

3. Acceptability of the study design will be measured by the number of primary care practices and patients who take part and remain in the trial (that is, appointment attendance and intervention, data and study completion rates) and reasons for drop-out, where provided will be examined. In a situation where a patient does not attend an appointment but contributes data at a specific data collection time point, this information will be recorded.

4. Primary care practice and patient recruitment rates will be measured by the number who consent to be randomised divided by the number eligible to be randomised and the number taking part in the trial divided by the length of the recruitment period. The practice recruitment period begins when the first round of practice invitations are mailed out and ends when the last practice recruited is randomised. Patient recruitment begins when the first group of eligible patients is mailed their invitation letter and ends when the last patient recruited provides informed written consent.

5. Willingness to take part in video recordings of consultations, semi-structured interviews and focus group discussions will be assessed in both the intervention and control groups.

6. Adherence to the study protocol (fidelity assessment) and barriers/facilitators to effective implementation of MaMT2D in the primary care setting will be assessed throughout the trial period. A proportion of consultations will be video recorded to assess adherence to intervention components; and data collection completion rates will be monitored and recorded at each data collection time point. Semi-structured interviews with participating primary healthcare professionals and patients will be conducted to identify barriers and enabling factors to implementation of the intervention in routine primary care.

### Secondary outcome measures

1. Estimates of variability will be calculated for objectively assessed PA behaviour and diabetes-related knowledge, attitudes/beliefs, self-efficacy for being physically active, health related quality of life, HbA1c, blood pressure, waist circumference and body mass index in patients at each data collection time point. Estimates of variability will include the standard deviation, range and interquartile range for each study outcome. Estimates of standard deviation will be based on an appropriate multilevel model (subjects nested within practices); the variance will be partitioned into between subject and between practice components.

2. Estimates of variability will be calculated for fidelity of specific behaviour change techniques, behaviour change counselling skills (in the context of diabetes management) and PA related knowledge, self-reported PA behaviour, attitudes/beliefs and self-efficacy for delivering a behavioural intervention.

3. A qualitative process evaluation will be undertaken concurrently to assess barriers and facilitators to effective implementation of the intervention into routine primary care. A refined and optimised version of both components of MaMT2D (online training intervention programme for primary healthcare professionals and the range of printed materials, DVD and pedometer (SW200, Yamax Corporation, Tokyo, Japan) for patients (as part of the primary healthcare professional-led intervention) will be compared with usual clinical care. Assessment of all outcomes will inform future work (for example, a definitive RCT).

### Patient outcome measures

PA behaviour will be measured objectively using a blinded (that is, no data visible to patients) wrist worn tri-axial accelerometer (GENEActiv, ActivInsights Ltd, Kimbolton, UK) for seven continuous days at each data collection time point. Clinical outcome measures, comprising glycaemic control (HbA1c), diastolic and systolic blood pressure (mmHg), waist circumference (cm) and body mass index (kg/m^2^), will be measured and recorded by primary healthcare professionals during review appointments. Self-reported PA (International Physical Activity Questionnaire (IPAQ)) [49]; diabetes-related knowledge (Audit of Diabetes Knowledge (ADKnowl)) [50]; behavioural intentions, subjective norms and attitudes/beliefs about PA for the management of diabetes (Physical Activity Questionnaire for Diabetic Patients (PAQ-DP)) [51]; PA-related self-efficacy [52]; and health-related quality of life (Audit of Diabetes Dependent Quality of Life (ADDQoL)) [53] will be measured by self-report postal questionnaire. Each outcome will be assessed at baseline, one, six and twelve-month follow up. Background information will be collected from patients on age, gender, employment status, length of time since diagnosis of Type 2 diabetes and method of diabetes control (that is, diet, oral medication).

### Healthcare professional outcome measures

Change in consultation behaviour involving utilisation of selected behaviour change techniques and behaviour change counselling techniques will be assessed subjectively using a questionnaire specifically developed for the purpose of this study. Objective assessments will be made by video recording a sub-sample of primary healthcare professionals in practice. Self-reported diabetes and PA-related knowledge; behavioural intentions, subjective norms, attitudes/beliefs about PA as a management option for Type 2 diabetes (adapted PAQ-DP) [51]; self-efficacy for delivering a behaviour change intervention [52]; and PA behaviour (IPAQ) [49] will be assessed by online questionnaire at each data collection time point (baseline, one, six and twelve months follow up). Background information will be collected about current job title and role, length of time in current role, details of any previous training undertaken in the area of health behaviour change (for example, behaviour change counselling and motivational interviewing), diabetes and PA for the management of Type 2 diabetes.

### Treatment fidelity assessment

A sub-sample (20% to 40%) of consultations will be video recorded to assess fidelity of primary healthcare professionals use of specific behaviour change techniques and behaviour change counselling techniques within MaMT2D consultations (and usual care consultations) with patients. Each intervention component will be coded as either ‘present’ or ‘absent ‘and an assessment will be made on the extent to which it was utilised (that is, how closely delivery meets pre-specified criteria of behaviour change techniques (BCTs) and intervention components). The Behaviour Change Counselling Index (BECCI) [54] will be used to assess primary healthcare professional’s use of behaviour change counselling skills to enhance patient-centeredness of consultations. A sub-sample of video recordings will be double coded by a second coder and any discrepancies will be resolved by a third coder. All coders will have expertise in theory-linked BCTs, including experience using the 40-item taxonomy [55] used to define BCTs and behaviour change counselling techniques included in MaMT2D. This assessment will enable quantification of primary healthcare professional and patient interactions, to provide insights into the therapeutic relationship, as well as allow an assessment to be made as to whether this relationship translates into actual PA behaviour change. The outcome of these analyses obtained during the pilot RCT will help to understand the nature of the interaction during MaMT2D consultations and usual clinical care, and to identify any potential barriers and facilitators to effective implementation of the intervention in routine primary care. Assessment of treatment fidelity will help determine whether any estimated differences detected in objectively assessed PA behaviour between the intervention and control group patients can be attributed to any changes in consultation behaviour of participating primary healthcare professionals (influenced by the online training intervention programme) [42,56].

To maximise treatment fidelity a number of strategies will be used.

### Treatment fidelity strategies for design of the study

Both intervention and control group patients will receive the same ‘treatment dose’ (that is, the same number of contacts from primary healthcare professionals and the research team). To plan for implementation setbacks a minimum of two primary healthcare professionals will be recruited (and trained in the intervention arm of the study) from each primary care practice. By utilising treatment fidelity strategies related to study design, the study aims and objectives can be adequately tested.

### Treatment fidelity strategies for monitoring and improving provider training

A standardised online training intervention programme will be provided to ensure all primary healthcare professionals in the intervention arm of the study receive access to the same training content. Where primary healthcare professionals do not adhere sufficiently to delivery of pre-selected behaviour change techniques and behaviour change counselling skills, the research team will explore possible reasons for this and changes to the intervention will be made as appropriate during the open pilot study. Video recordings of consultations will be made throughout phases one and two to assess skill acquisition, utilisation and treatment fidelity.

### Treatment fidelity strategies for monitoring and improving receipt of the intervention

A qualitative process evaluation and treatment fidelity assessment will be conducted throughout phases one and two to assess primary healthcare professional performance on delivery of specific behaviour change techniques and behaviour change counselling skills.

### Qualitative process evaluation

Interviews will be carried out with a purposive sample of primary healthcare professionals and patients during phases one and two of the study. Interview topic guides will be developed using the TDF [38]. The outcome of interviews will help to optimise the intervention, and streamline study processes and procedures during phase one and to obtain an in-depth understanding of barriers and facilitators to successful implementation during phase two. Interviews will be audio recorded, transcribed and data will be analysed using thematic analysis. A proportion of transcripts will be analysed by a second researcher.

### Data collection, storage and security

Data collected via audio recorders, video recorders and accelerometers will be saved to a password protected server in the Institute of Cellular Medicine (ICM) at Newcastle University. These data will be accessed only by members of the research team. Once all data have been uploaded to the server they will be securely deleted from the recording devices. Patient questionnaire data will be anonymised and stored in locked filing cabinets in the ICM.

### Randomisation and concealment of allocation

Practice recruitment will be carried out in two stages. Practices will be randomly allocated to the intervention or usual clinical care group after each recruitment stage and following receipt of informed written consent from participating primary healthcare professionals. Consent will be obtained by a member of the research team who is blind to details of randomisation sequencing and concealment. Practices will be allocated to one of four strata based on list size with three cut-off points: <3,280; ≥3,280 to 5,989; ≥5,990 to 10,453; and >10454 (thresholds based on quartiles of distribution of list size across all practices in the County Durham & Darlington area of North East England) and will be randomised independently within strata using random permuted blocks. The blocks will be generated using a subroutine that utilises the ‘rand()’ function built into the programming language awk implemented on the central Linux system at Newcastle University. The size of blocks will be concealed until the end of recruitment.

### Blinding

It is inherently difficult to blind primary healthcare professionals and patients in behavioural interventions with the nature of usual clinical care for diabetes being so well defined. Therefore, blinding of participants will not be possible. Due to pragmatic reasons researchers will not be blinded to group allocation of participating practices; however, the statistical analyses will be performed by a statistician blind to the treatment allocation of participating primary healthcare professionals and patients.

### Analyses

Analyses of data collected from phase two (the pilot RCT) will be descriptive with key outcomes being interval estimates of the variables of interest relating to assessment of acceptability, feasibility and data completeness. Although we may not have the power to detect a clinically important difference between groups we will generate interval estimates of the mean difference between groups for the main outcome measures to be included in a definitive trial (for example, PA and HbA1c). This will allow us to test the appropriateness of our analytic procedures. As this is a pilot study, the level of missing data will be documented but no imputation will be undertaken. Since we can not rule out the possibility of a very large difference between groups, the outcome of the analyses may suggest that a larger trial is not required. Planned exploratory sub-group analyses will be conducted to determine whether differences exist between primary healthcare professionals based on role and between patients as a function of gender, age, method of diabetes control and length of time since diagnosis of Type 2 diabetes.

### Ethical Approval

A favourable ethical opinion for this study (protocol version 1.1) was granted by Sunderland Research Ethics Committee (application number 12/NE/0037). National Health Service (NHS) Research & Development approval was granted by NHS County Durham & Darlington. The trial was subsequently adopted by the CLRN portfolio to receive support from both the Diabetes Research Network (DRN) and Primary Care Research Network (PCRN).

## Discussion

PA/exercise is an integral part of diabetes management. However, the use of PA/exercise in routine clinical care for adults with Type 2 diabetes is hindered by the lack of acceptable, feasible and robustly evaluated interventions. Availability of such interventions will enable diabetes care providers to support their patients to increase and maintain their levels of free-living PA that are sufficient for achieving long-term glycaemic control.

The effective delivery of behavioural interventions is compromised by the lack of evidence-based training programmes to equip healthcare professionals with a range of intervention-specific competencies (knowledge and skills) and confidence to support adults with Type 2 diabetes to become more physically active in the long-term. A systematic review of 17 trials reported use of treatment fidelity measures in a majority of studies in terms of study design (for example, consistent duration and frequency of sessions across trial arms) and monitoring to improve receipt of intervention components and enactment of intervention–related knowledge and skills by patients [11]. In contrast, treatment fidelity strategies to monitor and improve provider training and delivery of treatment in practice were frequently omitted (for example, use of standardised training materials, observation of intervention implementation during training and video recordings of sessions with intervention providers and recipients). Training of intervention providers and treatment fidelity assessment are important for ensuring reproducibility of intervention-related skills in clinical practice and increasing the likelihood of implementation in routine clinical care [43,56].

The current study aims to address the above three barriers to facilitate use of PA as a management option for Type 2 diabetes in the routine primary care setting. MaMT2D is, to the best of our knowledge, the UK’s first online accredited training intervention programme to support primary healthcare professionals to develop the competencies to effectively deliver a primary healthcare professional-led intervention targeting the increase and maintenance of free-living PA in patients with Type 2 diabetes.

MaMT2D has been developed through a structured participatory design process, involving healthcare professionals and adults with Type 2 diabetes in the primary care setting working alongside psychologists, physiologists, designers and clinical experts. The theoretical underpinning of the multifaceted behaviour change intervention is evidence-based and the content informed by a systematic review of the evidence on behaviour change strategies and other intervention features used in behavioural interventions targeting free-living PA in adults with Type 2 diabetes. The approach used to develop the intervention maximises the likelihood that MaMT2D is acceptable and feasible for use by primary healthcare providers and patients in the clinical setting.

An open pilot design will allow opportunities for optimisation and refinement of study procedures and information content of both the online training intervention (primary healthcare professionals) and paper-based materials in the patient toolkit for use within the primary healthcare professional-led intervention in accordance with the preferences of primary healthcare professionals and patients. A subsequent external pilot cluster RCT (informed by the findings of the open pilot) and concomitant qualitative process evaluation and fidelity assessment will assess to what extent intervention components can be reliably and faithfully delivered in routine practice. Interval estimates of the mean difference between groups for the main outcome measures at each time point will be reported. Treatment fidelity strategies (use of standardised training, study manuals, ongoing process evaluation and video recording and assessment of patient sessions (intervention and usual clinical care)) will be utilised throughout the intervention period to serve two main purposes: 1) to ensure the intervention is being delivered according to the protocol; and 2) to identify any barriers to successful delivery of the intervention content and implementation in routine practice.

## Trial status

Recruitment of primary healthcare practices to MaMT2D commenced during July 2012. At the time of manuscript submission, twelve practices had provided informed written consent to participate and eleven had been randomised.

## Abbreviations

BCT: behaviour change technique; CLRN: Comprehensive Local Research Network; CONSORT: consolidated standards of reporting trials; DRN: Diabetes Research Network; HbA1c: glycated hemoglobin A1c; MaMT2D: Movement as Medicine for Type 2 Diabetes; NHS: National Health Service; PA: physical activity; PCRN: Primary Care Research Network; RCT: randomised controlled trial; SCT: social cognitive theory; SPIRIT: standard protocol items: recommendations for intervention trials; TDF: theoretical domains framework; TPB: theory of planned behaviour.

## Competing interests

The authors declare that they have no competing interests.

## Authors’ contributions

MIT is the Chief Investigator and obtained research funding to develop and pilot the intervention and developed the physiological components of the MaMT2D online training intervention programme. LA developed the behavioural components of the online training intervention programme and the primary healthcare professional-led intervention that includes printed materials and patient DVD. LA, EMc, NS, FFS and MIT contributed to study design. Continuing clinical advice and expertise was provided by RT. LA will project manage the open pilot and pilot RCT on behalf of MIT. SJD will be responsible for collection and management of accelerometry data. LA and SJD will collect and analyse qualitative and quantitative study data. NS will provide statistical expertise, including analysis of trial data. EMc will provide clinical trial expertise. LA and SJD will write-up results for discussion with the study team. LA drafted the manuscript. All authors have read and approved the final manuscript.

## Supplementary Material

Additional file 1 SPIRIT Checklist.pdf. Click here for file

Additional file 2 Healthcare Professional Consent Form.pdf. Click here for file

Additional file 3 Practice Information Sheet.pdf. Click here for file

Additional file 4 Patient Information Sheet Control Group.pdf. Click here for file

Additional file 5 Patient Information Sheet Intervention Group.pdf. Click here for file

Additional file 6 Patient Consent Form.pdf. Click here for file

## Research dissemination strategy

The outcome of this trial will be disseminated to participating healthcare professionals and patients during a series of evening seminars. Trial results will be written up for publication and submitted to a peer reviewed journal. All authors will fulfil The International Committee of Medical Journal Editors (ICMJE) criteria for authorship.
