# Supplementary material for: Movement as Medicine for Type 2 Diabetes: protocol for an open pilot study and external pilot clustered randomised controlled trial to assess acceptability, feasibility and fidelity of a multifaceted behavioural intervention targeting physical activity in primary care
Source: Trials. 2014 Feb 3;15:46. doi: 10.1186/1745-6215-15-46 (PMC3922792; doi:10.1186/1745-6215-15-46)
Supplement: Additional file 2 — Healthcare Professional Consent Form.pdf. [file 1745-6215-15-46-S2.pdf]

Institute of Cellular Medicine, Newcastle University

Project title: Movement as Medicine for Type 2 Diabetes

Principal Investigator: Prof Mike Trenell, Newcastle University

Healthcare Professional Consent Form

Please initial

1. I confirm that I been fully informed about my involvement in the Movement as Medicine for Type 2 diabetes study. I have read and understood the Participant Information Sheet dated 12.03.2012 version 1.1 for the study and have had an opportunity to ask questions. ☐

2. I understand that my participation is voluntary and that I am free to withdraw at any time, without giving any reason. ☐

3. I agree take part in the above study. ☐

Name of Healthcare professional	Signature	Date
Researcher	Signature	Date
Name of Person taking consent (If different from researcher)	Signature	Date

1. I agree, that if selected I will allow up to four of my diabetes review appointments with each patient recruited to be video recorded for the purpose of this research. ☐

2. I agree, that if selected I will take part in an interview with a researcher from Newcastle University for the purpose of this research. ☐

3. I agree, that if selected I will take part in a focus group discussion with a researcher from Newcastle University and other primary care practitioners recruited to the study for the purpose of this research. ☐

____________________	____________________	____________________
Name of Healthcare professional	Signature	Date
____________________	____________________	____________________
Researcher	Signature	Date
____________________	____________________	____________________
Name of Person taking consent (If different from researcher)	Signature	Date

Practice Name:

Practice Identification Number:

Participant Identification Number:

1 copy for researcher; 1 copy for patient; 1 copy to be filed within medical record
