# Supplementary material for: Movement as Medicine for Type 2 Diabetes: protocol for an open pilot study and external pilot clustered randomised controlled trial to assess acceptability, feasibility and fidelity of a multifaceted behavioural intervention targeting physical activity in primary care
Source: Trials. 2014 Feb 3;15:46. doi: 10.1186/1745-6215-15-46 (PMC3922792; doi:10.1186/1745-6215-15-46)
Supplement: Additional file 3 — Practice Information Sheet.pdf. [file 1745-6215-15-46-S3.pdf]

Movement as Medicine for Type 2 Diabetes

Principal Investigator: Prof Mike Trenell, Newcastle University

Research Funded by:
NHS North East Health Innovation Education Cluster

Information Sheet for Healthcare Professionals

Thank you for taking time to read this information. You are being invited to take part in a research study about physical activity and Type 2 diabetes. Before you decide whether or not you would like to take part, it is important for you to understand why the research is being carried out and what it will involve. Please take time to read this information sheet and discuss it with others should you wish. Participation is entirely voluntary and if you do decide to take part, you are still free to withdraw at any time without giving a reason.

What is the purpose of the study?
Research has shown that increasing everyday levels of physical activity can produce significant improvements in blood glucose control in people with Type 2 diabetes. This can often bring about similar effects shown with some oral diabetes medications and has the potential to halt progression to other diabetes medications including insulin. What is less well understood is how best to support people with Type 2 diabetes to become more physically active and maintain this over time.

The research study you are being invited to take part in is called 'Movement as Medicine for Type 2 diabetes'. It aims to find out whether structured behavioural support delivered by a primary healthcare professional is feasible, acceptable and effective for increasing levels of physical activity in people with existing non-insulin dependent Type 2 diabetes. If you agree to take part your practice will be allocated at random to either the intervention group (usual clinical care plus structured behavioural support targeting physical activity plus a Diabetes UK leaflet) or the control group (usual clinical care plus a Diabetes UK leaflet). If your practice is randomised to the intervention group you will receive access to an evidence-based online accredited training programme designed to provide you with the knowledge and skills to deliver the intervention (structured behavioural support). We aim to recruit up to 40 practices from the County Durham and Darlington region and you have been invited to take part because your practice is located within this region.

Do I have to take part?
Participation is entirely voluntary; therefore it is up to you whether or not you decide to take part. If you agree to take part we ask that you sign and return the consent form enclosed with this information sheet. You are still free to withdraw at any time without giving a reason. It is important however that at least two healthcare professionals from your practice agree to participate to offer patients continuity of care if one member of your team decides to withdraw for any reason.

What will be in involved if I agree to take part?
Your practice will be allocated at random to either the intervention (usual clinical care plus structured behavioural support plus a Diabetes UK leaflet) or control group (usual clinical care plus a Diabetes UK leaflet). We ask each practice to identify all patients eligible to take part in the study and randomly select a sample with the aim of recruiting 30 patients per practice in to the study. Once eligible patients have been identified, a research nurse from the Diabetes Research Network will help co-ordinate recruitment, schedule appointments and collect data once consent has been obtained.

If your practice is randomised to the intervention group:
In addition to delivering usual clinical care we ask you to provide structured behavioural support targeting physical activity to each patient recruited in to the study. Each patient should also be given a Diabetes UK leaflet to take home. To help you deliver the structured behavioural support you will be given access to an online accredited training programme and supporting materials. This programme aims to equip you with the knowledge and skills required to deliver structured behavioural support to increase levels of physical activity in people with existing non-insulin dependent Type 2 diabetes. The training consists of eight distinct but interrelated modules with a dedicated interactive skills based module that will take you through the process of delivering the intervention. Upon completion of the training you will be given access to a link where you can print off a certificate confirming that you have completed the course. At this point you will use the materials provided with ongoing support from the online training programme (should you want it) during each consultation with patients recruited to the study.

If your practice is randomised to the control group:
In addition to delivering usual clinical care we ask you to give each patient recruited in to the study a Diabetes UK leaflet. You are not required to explain the leaflet or offer anything else in addition to their usual clinical care.

Intervention and control group practices:
We ask that you undertake four face-to-face diabetes review appointments with each patient recruited in to the study over a 12 month period. These should take place at baseline, 1 month, 6 months and 12 months. In addition each patient should receive a telephone call/motivational postcard at month 3 to offer support.

In addition we ask that every patient recruited in to the study has their HbA1c, body mass index, blood pressure and waist circumference measured and recorded at each review appointment.

Assessment of Movement as Medicine is in three parts for both intervention and control group practices
1. We ask you to complete an online questionnaire at the beginning of the study and again at 1, 6 and 12 months follow up.

2. We will ask a small number of healthcare professionals from intervention and control group practices if we can video record up to four diabetes review appointments with each participating patient. This will allow the research team to observe the delivery of the intervention and make any changes to the training programme where necessary to improve it. Observing video recordings of control group review appointments will allow the research team to fully understand the nature of usual clinical care. Video footage will be viewed by two researchers and observations compared. This aspect of the study is entirely voluntary and you can take part in the wider study without agreeing to this aspect of it. Video recordings will be used for the purpose of this research only and will not be shared with any third party including colleagues at your place of work. You will see from the consent form that we ask separately for consent to include you in this part of the study. If you are happy to participate please sign the appropriate section of the form.

3. You may be invited to take part in an interview and/or focus group discussion with a researcher. These will be carried out with intervention and control group practices and will allow the research team to understand what factors might affect the implementation of the Movement as Medicine programme in routine clinical care. Interviews and focus group discussions with the control group are designed to find out what aspects of usual clinical care you find works well (or not so well) to promote an increase in physical activity. We are particularly interested in the barriers and facilitators to supporting people with Type 2 diabetes to become more physically active in routine primary care. Interviews will last up to 30 minutes and focus groups up to 60 minutes. They will be audio recorded and transcribed verbatim. Again this part of the process is entirely voluntary and you can participate in the wider study without agreeing to this aspect of it. You will see from the consent form that we ask separately for consent to include you in this part of the study. If you are happy to participate please sign the appropriate section of the form.

What are the possible risks or disadvantages of taking part?
Participation time has to be considered. It is envisaged that the online training programme will take approximately two hours to complete (although this can be completed in smaller sections over a period of four weeks). In addition you will receive an online questionnaire to complete four times over the study period which will take approximately 20 minutes per questionnaire to complete. Each patient entering the study should receive four diabetes review appointments over a 12 month period.

What are the possible benefits of taking part?
Research has shown that providing evidence based structured behavioural support to people with Type 2 diabetes can help them to become more physically active and impact positively on blood glucose levels over time. Furthermore other benefits can be derived from being more physically active such as prevention of weight gain and improved mood. The online training programme provided via Movement as Medicine aims to provide you with the knowledge and skills necessary to deliver evidence based structured behavioural support using a toolkit developed for use with your patients. Furthermore, it is envisaged that the skills developed from this programme will be transferable to a range of clinical situations when addressing lifestyle behaviour change.

What data will be collected and how will it be used?
An online questionnaire will be used throughout this study to collect data on diabetes and physical activity-related knowledge, attitudes/beliefs, physical activity behaviour and confidence for delivering structured behavioural support targeting physical activity with your patients. This data and data collected via interviews and focus groups will be used to improve the intervention where (and if) necessary and to understand whether the intervention is any better than usual clinical care for increasing levels of physical activity in adults with Type 2 diabetes. Data collected from both the intervention and control groups will allow this comparison to be made. Furthermore, the research team will gain an understanding of what factors might facilitate or prevent the use of Movement as Medicine in routine primary care should it prove to be beneficial for healthcare professionals and patients.

Will the information I provide be kept confidential?
Any information you provide will be treated as confidential and will only be accessed by the research team. We are bound by very strict rules about the use of confidential information. Any information about you which leaves the research setting will have your name and practice details removed so that you cannot be recognised from it. Instead an identification number will be used.

In accordance with Newcastle University's policy on data protection and storage, all information you provide via questionnaire and transcripts of interviews and focus group discussions will have your name and other identifiable information removed. Questionnaires will be held securely on a password protected server accessed only by members of the research team. Any other electronic information will be stored

securely on password protected computers in the Institute of Cellular Medicine at Newcastle University. Again only members of the research team will have access to this information.

None of your personal information will be identified in any reports about the research. At the end of the study your personal information will be deleted.

What will happen if I change my mind about taking part?
If you agree to participate, but later decide to withdraw, please inform the principal investigator of this study or a member of his team. You are free to withdraw from the study at any time without providing a reason. Any information you contribute to the study up until the point you withdraw will be included in the analyses unless you specifically ask us to remove it. No further information will be collected from you once you withdraw.

What will happen to the results of the study?
We intend to publish the findings of the study in a report, scientific journals and present it at scientific meetings. Any information that could potentially identify you will not be included in any report, publication or presentation. You are welcome to have a copy of the results once they are published. The research team will hold an event for participants where they will present the findings of the study which you will be invited to attend.

Who is organising and funding the research?
The development of the Movement as Medicine intervention was funded by County Durham & Darlington Primary Care Trust (PCT). The piloting of the intervention is funded by the NHS North East Health Innovation Education Cluster and the Medical Research Council. The design and organisation of the study is the responsibility of Professor Trenell and his research team.

Has the study been approved by a research ethics committee?
The study has received a favourable opinion from the Sunderland Research Ethics Committee. They have looked closely at the research to ensure that participants will not be harmed as a result of the procedures being used and that confidentiality will be assured. The study has also been registered with a national research register and has been given a trial number: ISRCTN67997502.

What happens next?
If you decide you would like to take part in this study could you please complete and sign your consent form. Either a member of the research team or the Diabetes Research Network will arrange a time to countersign and collect your consent form and during this time you will be given an opportunity to ask any questions. Please feel free to discuss the study with colleagues before agreeing to take part. It is not essential that all healthcare professionals in your practice agree to participate, however it is important to consider that all patients recruited to the study will require support for the duration of the study. Should a member of your clinical team withdraw, another member of the team should be available to continue delivering care dictated by the study group to which you are allocated. It is therefore essential that at least two healthcare professionals consent to participate in the study. Once we receive completed consent forms from all members of your practice wishing to participate, your practice will be allocated at random to the intervention or control group. You will be informed of group allocation in due course. You will then receive a unique login ID to gain access to an online questionnaire and depending upon your practice allocation, access to the online training programme and supporting materials.

Who can I contact if I have any questions about the study or if I experience any problems while taking part?
Please feel free to ask us any questions about the study. The research team will be happy to explain anything that is unclear or address any concerns you have. If you wish to make a complaint about any aspect of this study it will be dealt with immediately by Professor Trenell.

Thank you for taking the time to read the information sheet. Please feel free to ask us any questions about the study or how it will be carried out. If you do decide to take part please keep this information sheet for future reference.

Queries about the study can be directed to the principal investigator:

Professor Mike Trenell,
Director, MoveLab; Physical Activity & Exercise Research
Institute of Cellular Medicine
4th Floor, William Leech Building
Newcastle University
Newcastle upon Tyne
NE2 4HH
michael.trenell@ncl.ac.uk
0191 2226935
