# Supplementary material for: Movement as Medicine for Type 2 Diabetes: protocol for an open pilot study and external pilot clustered randomised controlled trial to assess acceptability, feasibility and fidelity of a multifaceted behavioural intervention targeting physical activity in primary care
Source: Trials. 2014 Feb 3;15:46. doi: 10.1186/1745-6215-15-46 (PMC3922792; doi:10.1186/1745-6215-15-46)
Supplement: Additional file 4 — Patient Information Sheet Control Group.pdf. [file 1745-6215-15-46-S4.pdf]

Movement as Medicine for Type 2 Diabetes

Principal Investigator: Professor Mike Trenell, Newcastle University

Research Funded by:
NHS North East Health Innovation Education Cluster

Patient Information Sheet

Thank you for taking time to read this information. You are being invited to take part in a research study about physical activity and Type 2 diabetes. Before you decide whether or not you would like to take part, it is important for you to understand why the research is being carried out and what it will involve. Please take time to read this information sheet and discuss it with others should you wish. Participation is entirely voluntary and if you do decide to take part, you are still free to withdraw at any time without giving a reason.

What is the purpose of the study?
Research has shown that increasing everyday levels of physical activity can produce significant improvements in blood glucose control in people with Type 2 diabetes. This can often bring about the similar effects shown with some oral diabetes medications and has the potential to delay progression to other diabetes medications including insulin. What is less well understood is how best to support people with Type 2 diabetes to become more physically active and maintain this over time.

The research study you are being invited to take part in is called 'Movement as Medicine for Type 2 Diabetes'. It aims to find out whether the use of a new programme called 'Movement as Medicine' is better than usual clinical care for supporting people with Type 2 diabetes to become more physically active. If you agree to take part you will continue to receive your usual clinical care but will also be given an information leaflet published by Diabetes UK about diabetes and physical activity. We aim to recruit 400 adults from the County Durham and Darlington region who have a confirmed diagnosis of non insulin dependent Type 2 diabetes (i.e., diabetes controlled by diet and/or oral medication - not insulin) and who are not already participating in any other research. You have been invited to take part because you meet these criteria.

Do I have to take part?
Participation is entirely voluntary therefore it is up to you whether you decide to take part or not. If you agree to take part we ask that you sign and return the consent form sent to you with this information sheet. You are still free to withdraw at any time without giving a reason and without your medical care being affected.

What will be in involved if I agree to take part?
If you agree to take part in the study you will be asked to attend four diabetes review appointments over a 12 month period. Your review appointments will run in exactly the same way as usual, but in addition you will receive an information leaflet published by Diabetes UK.

The research process is in three parts:
1. We will ask you to complete a questionnaire before your first review appointment. At the same time we will also ask you to wear a physical activity monitor for 7 days and return it with your completed questionnaire in a pre-paid envelope. We will ask you to repeat this process one month after your first appointment and again at 6 and 12 months in to the study. During each review appointment you will have a blood test taken to measure your long-term blood glucose levels (HbA1c), your blood pressure, waist circumference and body mass index. There is no review appointment at 3 months, however we do ask that a healthcare professional from your practice contacts you by telephone to answer any questions you may have.

2. A small number of participants will be asked to have their review appointments video recorded. The video recordings will allow the research team to look at the support you receive during your diabetes review appointment. Participation in this part of the study is entirely voluntary. You can take part in the wider study without agreeing to this aspect of it. If you agree to this part of the study, up to four of your appointments will be video recorded and footage will be looked at by two researchers. These video recordings will be only be used for the purpose of this research and will not be shared with anyone else. You will see that the consent form asks separately for your permission to record your review appointments. If you are happy to take part please sign the appropriate section of the form. You may or may not be selected.

3. You may be invited to take part in an interview with a researcher and/or a focus group discussion with a researcher and other patients from your practice. Again this part of the study is entirely voluntary and you can take part in the wider study without agreeing to this aspect of it. The discussions that take place during the interviews and focus group discussions will allow us to find out what kind of support you currently receive during routine diabetes review appointments. We are particularly interested in what you find helpful (or not so helpful) about the support you receive to become more physically active. Interviews will last up to 30 minutes and focus group sessions up to 90 minutes. They will be audio recorded and typed up by a researcher. All information will be made anonymous so that you cannot be identified. You will see that the consent form asks separately for your permission to invite you to an interview and/or focus group discussion. If you are happy to take part please sign the appropriate section of the form. You may or may not be selected.

What are the possible risks or disadvantages of taking part?
Giving up time to participate has to be considered. The study involves attending four review appointments, completing four questionnaires and wearing a physical activity monitoring device four times over a 12 month period.

What are the possible benefits of taking part?
This study will provide you with the opportunity to offer suggestions about how your primary care practice can better support you and other people like you to increase everyday levels of physical activity. The data you provide will allow the research team at Newcastle University

to compare it to data given by patients in other practices recruited to the Movement as Medicine for Type 2 Diabetes Programme. Comparing the data will allow researchers to understand whether the new programme is any better than usual clinical care for supporting people with Type 2 diabetes to become more physically active. Therefore your participation is very valuable in this respect.

Will the information I provide be kept confidential?
Any information you provide by questionnaire will be treated confidentially and will only be accessed by the research team. Blood glucose test results taken for the purpose of this study and measures of blood pressure, waist circumference and body mass index will be anonymised and sent to a member of the research team by a research nurse working with your practice from the Diabetes Research Network. This information will also be recorded on your medical records. All of this information will be treated confidentially.

We are bound by very strict rules about the use of confidential information.
Any information about you that leaves your primary care practice will have your name and address removed so that you cannot be recognised from it. Instead your name will be replaced by an identification number.

None of your personal information will be identified in any reports about the research. At the end of the study your personal information will be deleted.

In accordance with Newcastle University's policy on data protection and storage, all information you provide via questionnaire and transcripts of interviews and focus group discussions will have your name and other identifiable information removed. Questionnaires will be kept in a locked filing cabinet and accessed only by members of the research team. Any electronic information will be securely stored on password protected computers in the Institute of Cellular Medicine at Newcastle University. Again only members of the research team will have access to this information.

What will happen if I change my mind about taking part?
If you agree to participate, but later decide that you no longer wish to take part in this study, please inform your primary care practice or the principal investigator of this study using the contact details below. You are free to withdraw from the study at any time, without giving a reason and without your medical care being affected. Any information you contribute to the study up until the point you withdraw will be included in the analyses unless you specifically ask us to remove it. No further information will be collected from you or your medical records once you withdraw.

What will happen to the results of the study?
We intend to publish the findings of the study in a report, scientific journals and present it at scientific meetings. Any information that could identify you will not be included in any report, publication or presentation. You are welcome to have a copy of the results once they are published. The research team will hold an event for participants where they will present the findings of the study which you will be invited to attend.

Who is organising and funding the research?
This research project is funded from a grant awarded by the NHS North East Health Innovation Education Cluster and the Medical Research Council. The design and organisation of the study is the responsibility of Professor Mike Trenell and his research team.

Has the study been approved by a research ethics committee?
The study has received a favourable opinion from the Sunderland Research Ethics Committee. They have looked closely at the research to ensure that participants will not be harmed as a result of the procedures being used and that confidentiality will be assured. The study has also been registered with a national research register and has been given a trial number: ISRCTN67997502.

What happens next?
If you decide you would like to take part in this study could you please complete, sign and return your consent form. You will then receive a questionnaire and physical activity monitoring device in the post with instructions on how to use it. We ask that you wear this for 7 days before returning it to us by post with your completed questionnaire in the prepaid envelope provided. You will also receive details of your first appointment. Please keep this information sheet in case you need to refer to it in the future.

Who can I contact if I have any questions about the study or if I experience any problems while taking part?
Please feel free to ask us any questions about the study. The research team will be happy to explain anything that is unclear or address any concerns you have. If you wish to make a complaint about any aspect of this study it will be dealt with immediately by Professor Mike Trenell.

If you would like to talk to somebody outside the research team about any concerns you have, you can contact the County Durham and Darlington Patient Advice and Liason Service (PALS) on their freephone number 0800 195 7998.

Professor Mike Trenell, PhD
Director, MoveLab; Physical Activity & Exercise Research
Institute of Cellular Medicine
4th Floor, William Leech Building
Newcastle University
Newcastle upon Tyne
NE2 4HH
michael.trenell@ncl.ac.uk
0191 2226935
