# Supplementary material for: Movement as Medicine for Type 2 Diabetes: protocol for an open pilot study and external pilot clustered randomised controlled trial to assess acceptability, feasibility and fidelity of a multifaceted behavioural intervention targeting physical activity in primary care
Source: Trials. 2014 Feb 3;15:46. doi: 10.1186/1745-6215-15-46 (PMC3922792; doi:10.1186/1745-6215-15-46)
Supplement: Additional file 5 — Patient Information Sheet Intervention Group.pdf. [file 1745-6215-15-46-S5.pdf]

Movement as Medicine for Type 2 Diabetes

Principal Investigator: Prof Mike Trenell, Newcastle University

Research Funded by:
NHS North East Health Innovation Education Cluster

Patient Information Sheet

Thank you for taking time to read this information. You are being invited to take part in a research study about physical activity and Type 2 diabetes. Before you decide whether or not you would like to take part, it is important for you to understand why the research is being carried out and what it will involve. Please take time to read this information sheet and discuss it with others should you wish. Participation is entirely voluntary and if you do decide to take part, you are still free to withdraw at any time without giving a reason.

What is the purpose of the study?
Research has shown that increasing everyday levels of physical activity can produce significant improvements in blood glucose control in people with Type 2 diabetes. This can often bring about similar effects shown with some oral diabetes medications and may slow down progression to other diabetes medications including insulin. What is less well understood is how best to support people with Type 2 diabetes to become more physically active and how to maintain this over time.

The research study you are being invited to take part in is called ‘Movement as Medicine for Type 2 Diabetes’. It aims to find out whether the use of structured support offered by a healthcare professional in primary care is feasible, acceptable and effective for increasing levels of physical activity in people with Type 2 diabetes. If you agree to take part you will receive support from a healthcare professional in primary care to become more physically active and ongoing support to maintain this over time. We aim to recruit 400 adults from the County Durham and Darlington region who have a diagnosis of non insulin dependent Type 2 diabetes (i.e., diabetes controlled by diet and/or oral medication - not insulin) and who are not already participating in any other research. You have been invited to take part because you meet these criteria.

Do I have to take part?
Participation is entirely voluntary therefore it is up to you whether you decide to take part or not. If you agree to take part we ask that you sign and return the consent form sent to you with this information sheet. You are still free to withdraw at any time without giving a reason and without your medical care being affected.

What will be in involved if I agree to take part?
If you agree to take part in the study you will be asked to attend four diabetes review appointments over a 12 month period. Your review appointments will run in exactly the same way as usual, but in addition to your usual care you will receive structured support from a healthcare professional to become more physically active. A ‘Movement as Medicine toolkit’ will be given to you to keep and that together with the support you receive at the practice will help you to plan and monitor your own activity levels over time. You will receive feedback on the progress you make and information about diabetes and the benefits of physical activity if and when you want it. The toolkit is made up of activity planners and trackers, an electronic device for monitoring your activity called a pedometer and an information DVD.

To allow the research team to assess the effect of Movement as Medicine, we will ask you to complete a questionnaire before your first review appointment. We will also ask you to wear a physical activity monitor for 7 days and return this to us with your completed questionnaire in a pre-paid envelope. We will ask you to repeat this process one month after your first appointment and again at 6 and 12 months in to the study. During each review appointment you will have a blood test taken to measure your long term blood glucose levels (HbA1c), your blood pressure, waist circumference and body mass index. There is no appointment scheduled at 3 months, however we do ask that a healthcare professional from your practice contacts you by telephone to ask how you are getting along and answer any questions you may have.

A small number of participants will be asked to have their review appointments video recorded. The video recordings will allow the research team to see how the programme is delivered to you and allow us to make any changes necessary to improve it. Participation in this part of the study is entirely voluntary (i.e., you can take part in the wider study without agreeing to this aspect of it). If you agree, up to four of your review appointments will be video recorded and footage will be looked at by two researchers. These video recordings will only be used for the purpose of this research and will not be shared with anyone else.

You may be invited to take part in an interview with a researcher and/or a focus group discussion with a researcher and other patients from your practice. Again this part of the study is entirely voluntary and you can take part in the wider study without agreeing to this aspect of it. The discussions that take place during the interviews and focus group discussions will allow us to find out what sort of things might affect the use of structured support targeting physical activity in routine practice. We are particularly interested in what went well (or not so well) with the programme, and where, if any, improvements could be made. Interviews will last up to 30 minutes and focus group sessions up to 90 minutes. They will be audio recorded and typed up by a researcher. All information will be made anonymous so that you cannot be identified. You will see that the consent form asks separately for your permission to invite you to an interview and/or focus group discussion. If you are happy to take part please sign the appropriate section of the form. You may or may not be selected.

What are the possible risks or disadvantages of taking part?
Giving up time to participate has to be considered. The study involves attending four review appointments, completing four questionnaires and wearing a physical activity monitoring device four times during the 12 month study period. There may also be a small chance, as with any study promoting an increase in physical activity that you may experience injury. However you will be supported to select for yourself a way to increase your everyday activity and you will be monitored to make sure this isn’t causing any harm.

What are the possible benefits of taking part?
Being more physically active has shown to have beneficial effects on long term blood glucose (HbA1c). Improvements in blood glucose levels may help to prevent diabetes related complications and being more physically active can bring about other benefits such as prevention of weight gain and improved mood.

Will the information I provide be kept confidential?
Any information you provide by questionnaire will be treated confidentially and will only be accessed by the research team. Blood test results and measures of blood pressure, waist circumference and body mass index will be recorded on your medical records and treated confidentially.

We are bound by very strict rules about the use of confidential information.
Any information about you that leaves your primary care practice will have your name and address removed so that you cannot be recognised from it. Instead your name will be replaced by an identification number.

None of your personal information will be identified in any reports about the research. At the end of the study your personal information will be deleted.

In accordance with Newcastle University's policy on data protection and storage, all information you provide via questionnaire and transcripts of interviews/focus groups you participate in will have all names and other identifiable information removed. Questionnaires will be kept in a locked filing cabinet and accessed only by members of the research team. Any electronic information will be securely stored on password protected computers in the Institute of Cellular Medicine at Newcastle University. Again only members of the research team will have access to this information.

What will happen if I change my mind about taking part?
If you agree to participate, but later decide that you no longer wish to take part in this study, please inform a healthcare professional from your practice or the principal investigator of this study using the contact details at the end of this sheet. You are free to withdraw from the study at any time, without giving a reason and without your medical care being affected. Any information you contribute to the study up until the point you withdraw will be included in the study unless you specifically ask us to remove it. No further information will be collected from you or your medical records once you withdraw.

What will happen to the results of the study?
We intend to publish the findings of the study in a report, scientific journals and present it at scientific meetings. Any information that could identify you will <u>not</u> be included in any report, publication or presentation. You are welcome to have a copy of the results once they are published.

Who is organising and funding the research?
This research project is funded from a grant awarded by the NHS North East Health Innovation Education Cluster and the Medical Research Council. The design and organisation of the study is the responsibility of Professor Trenell and his research team.

Has the study been approved by a research ethics committee?
The study has received a favourable opinion from the Sunderland Research Ethics Committee. They have looked closely at the research to ensure that participants will not be harmed as a result of the procedures being used and that confidentiality will be assured. The study has also been registered with a national research register and has been given a trial number: ISRCTN67997502.

What happens next?
If you decide you would like to take part in this study could you please complete and return your consent form. You will then receive a questionnaire and physical activity monitoring device in the post with instructions on how to use it. We ask you to wear this for 7 days and then return it to us by post with your completed questionnaire in the prepaid envelope provided. You will also receive details of your first appointment. Please retain this information sheet in case you need to refer to it in the future.

Who can I contact if I have any questions about the study or if I experience any problems while participating in the study?
Please feel free to ask us any questions about the study. The research team will be happy to explain anything that is unclear about the project or address any concerns you have. If you wish to make a complaint about any aspect of this study it will be dealt with immediately by Professor Mike Trenell.

If you would like to talk to somebody outside the research team about any concerns you have, you can contact County Durham and Darlington's Patient Advice and Liason Service (PALS) on free phone 0800 195 7998.

If you do have any queries about the study, they can be directed to the principal investigator:

Professor Mike Trenell, PhD
Director, MoveLab; Physical Activity & Exercise Research
Institute of Cellular Medicine
4th Floor, William Leech Building
Newcastle University
Newcastle upon Tyne
NE2 4HH
michael.trenell@ncl.ac.uk
0191 2226935

Or a member of his research team:

Please call: 0191 222 8264 and ask for Leah Avery or Sarah Denton

Thank you for taking the time to read this information.
