# Supplementary material for: Movement as Medicine for Type 2 Diabetes: protocol for an open pilot study and external pilot clustered randomised controlled trial to assess acceptability, feasibility and fidelity of a multifaceted behavioural intervention targeting physical activity in primary care
Source: Trials. 2014 Feb 3;15:46. doi: 10.1186/1745-6215-15-46 (PMC3922792; doi:10.1186/1745-6215-15-46)
Supplement: Additional file 6 — Patient Consent Form.pdf. [file 1745-6215-15-46-S6.pdf]

Institute of Cellular Medicine, Newcastle University and
County Durham & Darlington Primary Care Trust

Project title: Movement as Medicine for Type 2 Diabetes

Principal investigator: Prof Mike Trenell, Newcastle University

Patient Informed Consent Form

Please initial

1. I confirm that I been fully informed about my involvement in this study. I have read and understood the Information Sheet (Version 1 dated 20th January 2012) provided for the Movement as Medicine for Type 2 diabetes study and have had the opportunity to ask questions. ☐

2. I understand that my participation is voluntary and that I am free to withdraw at any time, without giving any reason. ☐

3. I understand that relevant sections of my medical notes and data collected during the study may be looked at by individuals from the NHS Trust/ Newcastle University Research Team and Regulatory Authorities, where it is relevant to my taking part in this research. I give permission for these individuals to have access to my records. ☐

4. I agree to take part in the Movement as Medicine for Type 2 diabetes study. ☐

Name of Patient: ____

Date: ____________________ Signature: __________________________

Researcher: _______________________ Date: ___________Signature: __________

_______________________ Date: __________ Signature: ___________
Name of Person taking consent
(If different from researcher)

1. I agree, that if selected I will allow up to a maximum of four of my diabetes review appointments with a primary care practitioner to be video recorded for the purpose of this research. ☐

2. I agree, that if selected I will take part in an interview with a researcher from Newcastle University for the purpose of this research. ☐

3. I agree, that if selected I will take part in a focus group discussion with a researcher from Newcastle University and other participants recruited to the study for the purpose of this research. ☐

Name of Patient: ____

Date: _______________________ Signature: _____________________________

Researcher: __________________________ Date: ____________Signature: ___________

__________________________ Date: ___________ Signature: ____________
Name of Person taking consent
(If different from researcher)

Practice Name:

Practice Identification Number:

Patient Identification Number:

1 copy for researcher; 1 copy for patient; 1 copy to be filed within medical record
